# Mapping anhedonia onto reinforcement learning: a behavioural meta-analysis

**DOI:** 10.1186/2045-5380-3-12

**Published:** 2013-06-19

**Authors:** Quentin JM Huys, Diego A Pizzagalli, Ryan Bogdan, Peter Dayan

**Affiliations:** 1 Gatsby Computational Neuroscience Unit, UCL, London, UK; 2 Wellcome Trust Centre for Neuroimaging, UCL, London, UK; 3 Translational Neuromodeling Unit, University of Zurich and ETH Zurich, Switzerland; 4 Department of Psychiatry, Psychotherapy and Psychosomatics, University Hospital of Psychiatry, Zurich, Switzerland; 5 Department of Psychiatry, Harvard Medical School, MA, USA; 6 Department of Psychology and Neuroscience, Duke University, NC, USA

**Keywords:** Anhedonia, Major depressive disorder, Depression, Reinforcement learning, Reward learning, Prediction error, Computational, Meta-analysis, Reward sensitivity, Learning rate

## Abstract

**Background:**

Depression is characterised partly by blunted reactions to reward. However, tasks probing this deficiency have not distinguished insensitivity to reward from insensitivity to the prediction errors for reward that determine learning and are putatively reported by the phasic activity of dopamine neurons. We attempted to disentangle these factors with respect to anhedonia in the context of stress, Major Depressive Disorder (MDD), Bipolar Disorder (BPD) and a dopaminergic challenge.

**Methods:**

Six behavioural datasets involving 392 experimental sessions were subjected to a model-based, Bayesian meta-analysis. Participants across all six studies performed a probabilistic reward task that used an asymmetric reinforcement schedule to assess reward learning. Healthy controls were tested under baseline conditions, stress or after receiving the dopamine D_2_ agonist pramipexole. In addition, participants with current or past MDD or BPD were evaluated. Reinforcement learning models isolated the contributions of variation in reward sensitivity and learning rate.

**Results:**

MDD and anhedonia reduced reward sensitivity more than they affected the learning rate, while a low dose of the dopamine D_2_ agonist pramipexole showed the opposite pattern. Stress led to a pattern consistent with a mixed effect on reward sensitivity and learning rate.

**Conclusion:**

Reward-related learning reflected at least two partially separable contributions. The first related to phasic prediction error signalling, and was preferentially modulated by a low dose of the dopamine agonist pramipexole. The second related directly to reward sensitivity, and was preferentially reduced in MDD and anhedonia. Stress altered both components. Collectively, these findings highlight the contribution of model-based reinforcement learning meta-analysis for dissecting anhedonic behavior.

## Background

Anhedonia is one of the cardinal symptoms for a clinical diagnosis of major depressive disorder (MDD; [1-3]) and refers to an inability to experience pleasure or a diminished reactivity to pleasurable stimuli. It is typically measured by verbal reports. In people subjectively reporting anhedonia, reward feedback objectively has less impact in a variety of behavioural tasks [4-15]. However, modern accounts of decision-making distinguish structurally different ways in which this reduction might be realized, and precisely which of these is associated with anhedonia remains unclear.

Here, we attempt to distinguish two critical factors. The first factor is a reduction in the *primary sensitivity* to rewards. This is possibly the closest behavioural equivalent to the notion of a reduction in consummatory pleasure. Instruments measuring anhedonia, such as the relevant subscores of the Beck Depression Inventory [16] or the Mood and Anxiety Symptom Questionnaire (MASQ) [17] typically focus on this factor [18]. The second factor is an alteration in participants’ ability to *learn* from reward feedback. This is emphasized by preclinical animal models of depression [19-22] and, because of the close association between dopamine (DA) signalling and reward learning [23-27], by neurobiological accounts linking anhedonia to DA [11,14,28-35]. It is most important to separate these factors, since they are likely to be associated with radically different ætiologies and therapeutic routes.

The distinction between these two factors is sharp in the mathematical formulation of reward learning based on prediction errors that underpins the account of DA activity [23-27], and is itself based on principles articulated in psychological [36] and engineering [37,38] theories of learning. Consider an experiment in which a reward of a given magnitude is given stochastically on some trials: where *r*_*t*_=1 if the participant did receive the reward on trial *t*, and *r*_*t*_=0 if it did not. We write *ρ* for the value the participant assigns to this reward. The participants are assumed to build and maintain an expectation ($Q_{t}$) of the average reward it might gain on this trial, by means of a prediction error on trial *t* which is the difference $\delta_{t}=\rho r_{t}-Q_{t}$ between the obtained *ρr*_*t*_ and expected $Q_{t}$ reward. This prediction error can be used to improve the expectations adaptively [39] by adding the error to the previous expectation $Q_{t+1}=Q_{t}+\epsilon \delta_{t}$, where 0≤*ϵ*≤1 is a learning rate. This reduces the prediction error over time, at least on average.

The critical factors that might be associated with anhedonia are the two parameters *ρ* and *ϵ* in the above expressions. First, *ρ* is a measure of primary reward sensitivity – the larger *ρ*, the more sensitive the participant is to a reward of a given magnitude, or the greater the internal worth of an external reward. We comment on the difference between liking and wanting rewards later [40]. Second, the term *ϵ* governs how reward prediction errors influence learning. Evidence suggests that the phasic activity of DA cells is roughly proportional to *δ*_*t*_; thus, alterations in the amount of DA released per spike, or in the sensitivity of postsynaptic receptors should behave like a change in the *learning rateϵ* and affect the speed at which reward affects behaviour [41].

**Figure 1:**
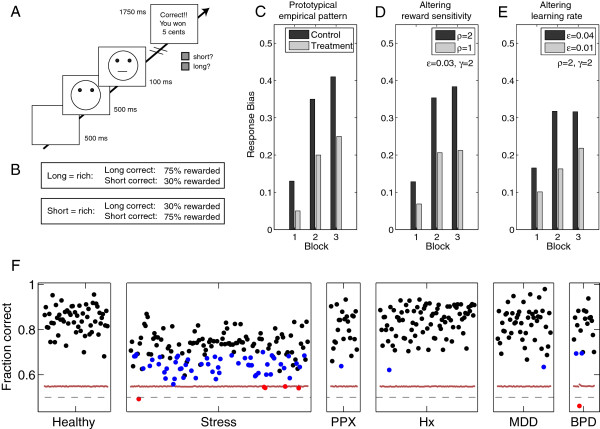
**Task and typical behaviour.A**: Task. Each trial had the following structure: 1) 500 ms presentation of a central fixation cross; 2) 500 ms presentation of face without a mouth; 3) 100 ms presentation of long (13 mm) or short (11.5 mm) mouth inside the face; 4) participants reported whether the mouth was long or short by key-press (‘Z’ or ‘/’ on US keyboard, counterbalanced); 5) Face without mouth remained on screen until participant response. Short and long stimuli were each presented 50 times per block in pseudorandom sequence avoiding more than three repetitions in a row. Adapted from [10]. **B**: Reward schedule. One response (counterbalanced across participants) had a higher reward expectation. Correct identification of that “rich” stimulus was more likely to be rewarded (75% probability) than correct identification of the other, “lean”, stimulus (30% probability). There was no punishment. If in doubt, choosing the more rewarded stimulus was beneficial. **C**: Surrogate simulated data showing prototypical response evolution. The dark bars show a hypothetical control group, developing a strong response bias towards the more rewarded response over the three blocks of 100 trials. The light bars show a prototypical treatment group with a reduced response bias. **D-E**: Surrogate simulated data generated from a simple reinforcement learning (‘Stimulus-action’) model. Both a reduction in reward sensitivity (**D**) and a reduction in learning rate (**E**) can roughly reproduce the pattern in the data (**C**). **F**: Percent correct responses for each of the 392 experimental sessions. Each black point represents one experimental session. Vertical bars demarcate datasets. Red horizontal line represents chance performance for each session. Four participants performed below chance (red). Sixty-three out of 392 experimental sessions were not fitted better than chance by model ‘Belief’ (binomial test; blue). Of these, 58 out of 63 were in the Stress dataset, in which performance was generally worst.

These quantities are formal parameters of a reinforcement learning rule. The question thus arises whether they can actually be distinguished in experimental practice. In this paper, we focus on objective measures of learning behavior. Crudely, since *ρ* only affects one term in *δ*_*t*_, whereas *ϵ* controls the effect of the whole of *δ*_*t*_ on $Q_{t+1}$, these quantities are theoretically distinguishable. A change in reward sensitivity leads to the asymptotic average value of $Q_{t}$ being different. However, a change in learning rate alters the speed at which this asymptote is reached. More particularly, if the behavioural impacts of anhedonia were due to a DA deficit, the extent of anhedonic symptoms might correlate preferentially with the learning rate *ϵ*. If instead its effects were due to a change in primary reward sensitivity, then one would expect the impact to concentrate on the reward sensitivity *ρ*. To examine this, we re-analyzed what is perhaps the most substantial body of data on reward learning in anhedonia, namely the probabilistic reward task of (Figure 1A-B). Behaviour in this task has previously been quantified by dividing it into three blocks and examining the evolution of a response bias across the blocks (Figure 1C). However, such measures cannot easily disentangle *ρ* and *ϵ*: Figure 1D-E shows that varying both parameters can roughly qualitatively explain the observed patterns. However, there are subtle differences to do with the asymptote of learning that provide us with a window of opportunity.

The fact that varying either parameter can lead to similar qualitative patterns shows that the two parameters play partially replaceable roles and may not be fully separable [42]. Any separation is likely to require substantial amounts of data. We therefore here fitted trial-by-trial individual reinforcement learning (RL) models in a meta-analytic manner to a series of six experiments involving 392 experimental sessions across 313 different participants. Model fitting allows for comprehensive tests of the ability of each hypothesis to account for the entire dataset. Bayesian model comparison ensures that the conclusions are based on parsimonious accounts of the data, avoiding overfitting and overly complex explanations [42-46].

To maximise the chance of identifying specific contributions of learning rate and reward sensitivity, we jointly analysed a series of datasets that are likely to differentially affect the two parameters (Table 1). Three of these probed anhedonia in the context of depression; one in bipolar disorder. A further dataset probed the effect of a dopaminergic manipulation, which we expected to primarily affect *ϵ*. A final dataset probed the effect of stress, which is prominently involved in the pathogenesis of depression, and which we have previously suggested may reduce phasic DA bursts via an increase of tonic DA release [47].

**Table 1:** Datasets used

**Name**	**Manipulation**	**Reference**	**Participants #**
Healthy	High *vs* low BDI scores	[10]	57
	Healthy volunteers. Payment: US$5 and course credit.		
MDD	MDD *vs* controls	[48]	48
	23 participants during an episode of MDD and 25 controls matched for age, sex, education, ethnicity and marital status. MDD participants met DSM-IV criteria for MDD, had Hamilton Rating Scale for depression scores ≥ 17, and no other axis I comorbidities except for anxiety. Inclusion required a minimal drug-free period of 2 weeks. Payment: US$10/hour plus 5US$ average task earnings.		
Hx	History of MDD *vs* no history	[49]	85
	Currently healthy participants with and without a history of major depressive disorder (MDD). Participants received US$15/hr in compensation for their time, as well as their task “earnings” (on average, US$5).		
BPD	BPD *vs* controls	[50]	19
	Euthymic outpatients (matched to the same 25 controls as in dataset ‘MDD’). The outpatients had a long-standing diagnosis of Bipolar Disorder, currently satisfying criteria on the Affective Disorder Evaluation, which contains modified SCID mood and psychosis modules. Patients were classified as euthymic if they currently Young Mania Rating Scale [51] score <12, and if their Hamilton Rating Scale for Depression [52] score was below 8. Exclusion criteria included other axis I disorders, a past history of MDD, substance abuse and ECT in the past 6 months. Participants were paid US$25 for participation and task earnings.		
PPX	Pramipexole *vs* placebo	[53]	24
	Healthy volunteers received either placebo or a single dose of the D2/D3 agonist pramipexole hydrochloride (PPX) 0.5 mg 2 hours prior to the task. At this low dose, PPX is thought to reduce phasic DA release through autoreceptor stimulation. Payment: US$ 40 for the pharmacological session and US$24.60 for the task session.		
Stress	Threat-of-shock acute *vs* no stressor	[54]	79(x2) +1
	Healthy volunteers took part in the task twice (one missing session), once in a no-stress condition and once in a stress condition. Participants were told that poor performance on the task might lead to a shock being delivered through electrodes attached to the back of their neck. In the stress condition, they were told that this was quite likely, whereas they were told that no shock would be delived in the no-stress condition. No shocks were actually delivered. Notably, the version of the task used in this study was more difficult, with the difference in size between long and short mouth being smaller. This resulted in fewer correct discriminations (see Figure 1F). Payment: either course credit or US$10/hour as well as money “won” during the task (US$10.60 on average).		

Full details of all the patient and control groups are provided in the original publications.

Our main result is that measures of anhedonia and depression preferentially affected the reward sensitivity *ρ*, while a dopamine manipulation by pramipexole mainly affected the learning rate *ϵ*. Stress, however, affected both reward sensitivity and the learning rate. There was no difference in these two parameters in euthymic bipolar individuals, or those with a past history of depression.

## Methods

### Task and data

In this paper, we re-analyse 392 sessions of behavioural data derived from a probabilistic reward task ([10]; adapted from [55]), which is displayed schematically in Figure 1A. Central to the task is that an asymmetrical reinforcement scheme was used to induce a response bias: Correct responses to one stimulus, designated “rich”, were more likely to be rewarded than correct responses to the other stimulus, designated “lean” (Figure 1B). No feedback was given on other trials, including incorrect trials, and no explicit information about the asymmetry was provided. Participants were explicitly encouraged to win as much money as possible, and so could benefit from reporting the rich, rather than the lean, stimulus when in doubt. One measure of the tendency to do this is the response bias [10]:

(1) $$\begin{array}{ll} \frac{1}{2}\log\left(\frac{n(a_{1}|s_{r})\;n(a_{1}|s_{l})}{n(a_{2}|s_{r})\;n(a_{2}|s_{l})}\right) & \; \end{array}$$

where *s*_*r*_ and *s*_*l*_ indicate presentation of the rich and lean stimulus, respectively, *a*_1_ and *a*_2_ are the two possible key presses, and *n*(*a*|*s*) is the number of times a particular choice was made in response to that stimulus. Each count *n* was augmented by $\frac{1}{2}$ to avoid numerical instabilities. Outlier trials with very short (<150 ms) or very long (>1500 ms) reaction times are excluded (see [10] for a full description of the 2-step procedure used to exclude trials with outlier responses). Figure 1F shows the fraction of correct responses for each of the 392 individual experimental sessions. In addition to the computer task, participants completed self-report questionnaires (see Table 2). The datasets and manipulations are shown in Table 1. Briefly, the studies examined the effect of i) depression (categorical diagnosis according to DSM-IV; continuous quantification based on self-report measures of depressive features and anhedonia; and past history of MDD); ii) bipolar disorder, currently euthymic (categorical diagnosis according to DSM-IV); iii) stress; and iv) low-dose D _2_ agonist pramipexole. The low dose (0.5 mg) of pramipexole was assumed to reduce phasic DA bursts to unexpected rewards due to presynaptic (autoreceptor) activation [53,56]. We note that the dataset ‘Stress’ differs from the others because a more difficult version of the task was used [54].

**Table 2:** Sample characteristics: psychometric measures

**Measure**	***N***	**mean**	**median**	**1st quartile**	**3rd quartile**
BDI	366	8.3	6	1	11
BDA	281	1.9	0	1	3
BDI\A	281	6.9	1	5	10
Generalized distress depression (GDD)	276	23.2	16	20	30
Generalized distress anxiety (GDA)	276	18.8	15	18	24
Anxious anxiety (AA)	276	53.7	45	53.5	65
Anhedonic Depression (AD)	276	21.1	17	20.5	25
Full (BDI + BDA + MASQ subscores)	255				

BDI is the total Beck Depression Inventory II [16]. BDA stands for the anhedonic subscore of the BDI consisting of the sum of items 4 (loss of pleasure), 12 (loss of interest), 15 (loss of energy) and 21 (interest in sex). BDI\A is the total BDI score without the anhedonic component (BDI\A = BDI - BDA). Mood and Anxiety Symptom Questionnaire (MASQ) subscores [17] were anxious arousal (AA), anhedonic depression (AD), general distress anxiety (GDA) and generalised distress depression (GDD).

### Reinforcement learning models

Reinforcement learning models account for every choice on every trial for every participant individually. Here, we describe the model for one particular participant. ‘Weights’ for emitting a particular choice are updated after every trial to predict the next choice. We consider a set of factors that might affect the weights, and use complexity-sensitive model comparison methods to try to identify the importance of each. Briefly, write *a*_*t*_ for the participant’s choice on trial *t* (key ‘/’ or ‘z’), and ${ā}_{t}$ for the choice not taken on that trial (key ‘z’ or ‘/’). If stimulus *s*_*t*_ (long or short mouth) was presented, the model assigns to *a*_*t*_ a probability *p*(*a*_*t*_|*s*_*t*_). This probability depends on the ‘weights’ $W_{t}(a_{t}|s_{t})$ and $W_{t}({ā}_{t}|s_{t})$ assigned to each choice when presented with stimulus *s*_*t*_. The mapping from weight to probability is made via a ‘softmax’ function so that a choice *a*_*t*_ will be expected to be emitted more frequently the bigger the difference between its weight and the weight of the alternative choice, or more specifically:

(2) $$p(a_{t}|s_{t})=\frac{1}{1+\exp\left[-(W_{t}(a_{t},s_{t})-W_{t}({ā}_{t},s_{t}))\right]}$$

The choice weights themselves change over time (hence the subscript on W) and are composed of several terms, whose contributions differ for the different models. The models are variants on an underlying full model called ‘Belief’, for which

(3) $$W_{t}(a_{t},s_{t})=\gamma I(a_{t},s_{t})+\zeta \;Q_{t}(a_{t},s_{t})+(1-\zeta )\;Q_{t}(a_{t},{\bar{s}}_{t})$$

The first of these terms, $\gamma I(a_{t},s_{t})$, depends on instructions: $I(a_{t},s_{t})=1$ if *a*_*t*_ is the instructed choice for stimulus *s*_*t*_ (for instance pressing ‘z’ for the long mouth) and is zero otherwise. The parameter *γ* thus determines the participants’ ability to follow the instructions. The bigger *γ*, the larger the contribution from $I(a_{t},s_{t})$, and hence the instructed response contributes more to choice. Importantly, this instructed choice is symmetric between rich and lean stimulus; thus this term leaves the asymmetry to the other terms.

The second and the third term depend on the expected reward $Q_{t}(a_{t},s_{t})$. This captures the effect of the experienced rewards on previous trials, just as described in the introduction (except allowing different predictions for the different actions and stimuli). $Q_{t}(a_{t},s_{t})$ depends on four factors: the binary sequence *r*_*t*_ up to that point in time, which indicates whether a reward was delivered or not, an initial $Q_{0}$ value, the learning rate *ϵ* and the subjective (i.e. internal to the participant as opposed to the external magnitude in a fixed number of cents) effect size of a reward *ρ*, which we identify with reward sensitivity.

After every choice, this Q value is updated according to the prediction error $\delta_{t}=\rho r_{t}-Q_{t}(a_{t},s_{t})$ as follows:

(4) $$Q_{t+1}(a_{t},s_{t})=Q_{t}(a_{t},s_{t})+\epsilon \delta_{t}$$

That is, after every trial, the expected reward Q(a,s) for choice *a* for stimulus *s* is increased towards the subjective reward size *ρ* if a reward is received (*r*_*t*_=1) but the expectation Q was lower than *ρ*, and it is decreased towards zero if no reward was received (*r*_*t*_=0). The larger *ρ*, the larger the effect of rewards on choice propensities. As the learning rate *ϵ* approaches 1, learning is so fast that the Q values are simply the last experienced outcome for each choice-action pair. For 0<*ϵ*<1, expectations represent exponentially weighted averages over the recent outcome history. A multiplicative change to *δ* is equivalent to a change in *ϵ*.

In the task, the mouth is only shown for a very short period of time. Thus participants cannot be sure which stimulus was actually presented, and, as experimenters, we cannot know what the participants perceived. This uncertainty has two consequences. First, it implies that the factor *γ* which governs the effect of the instructions, should be less than *∞*. Second, the participants will not be sure which value $Q_{t}(a_{t},\text{long})$ or $Q_{t}(a_{t},\text{short})$ and instruction weight $I(a_{t},s_{t})$ to employ in their choice, or which Q value to update using Equation 4. We capture this effect by assuming that they *know* which stimulus-choice pair to update in terms of learning (Equation 4), but that when choosing, they use a form of Bayesian decision theory [57] to combine estimates based on both possibilities. That is, we use a parameter 0≤*ζ*≤1 to represent participants’ average uncertainty about which stimulus was actually presented. Assume participants expected.75 unit reward for pressing button ‘z’ given the long mouth (Q(z,long)=0.75), and 0 given the short stimulus (Q(z,short)=0). If they now believed with a probability *ζ* that they had seen stimulus *s* and with a probability 1−*ζ* that they had seen stimulus $\bar{s}$, then their expectation for pressing button ‘z’ would be ζQ(z,long)+(1−ζ)Q(z,short)=.75ζ. This is the contribution of Q in Equation 3. We write *ζ* as applying to the Q term only. We could also apply it to the instruction term I(a,s), but this would be equivalent to rescaling *γ* (see Additional file 1).

Equations 3 and 4 comprise the full model ‘Belief’. We also considered two simpler variants, both of which had one fewer free parameter, and one more complicated variant, with an extra parameter. First, at *ζ*=1, participants are certain, and use the correct stimulus-action value to guide their choice. The model in which this value is forced is called ‘Stimulus-action’. At *ζ*=0.5, they are indiscriminate between the two stimuli. This is model ‘Action’ because they learn only about the values of choices, independently of stimuli. Finally, the more complex model ‘Punishment’ is based on the possibility that participants might treat a non-reward as a punishment, so making $\delta_{t}=\rho r_{t}+\rho^{-}(1-r_{t})-Q_{t}(a_{t},s_{t})$, where *ρ*>0. We mention other possible models in the discussion.

### Model fitting & comparison

Bayesian model comparison at the group level and model fitting procedures are described in detail in [58]. The key equations are provided in the Additional file 1. Briefly, model fitting was performed by Expectation-Maximisation to find group priors and individual (Laplace) approximate posterior distributions for the estimates for each parameter for each participant. For models ‘Action’ and ‘Stimulus-action’, this comprised parameters $\left\{Q_{0},\rho ,\epsilon ,\gamma \right\}$; for model ‘Belief’ it additionally included parameter *ζ*, and for model ‘Punishment’ also *ρ*^−^. All parameters were represented as non-linearly transformed variables with support on the real line and normally distributed group priors.

More complex models will often fit the data better because they have more freedom. However, model complexity is better assessed by methods other than counting parameters [43,59]. Bayesian model comparison is a principled way of assessing model parsimony by computing the posterior probability of each model given the entire dataset for all participants. Because exact computation of these quantities is intractable, individual parameters were integrated out by sampling from the fitted priors, and a standard Bayesian information criterion (BIC)-like approximation was employed at the group level [43]. This procedure results in a measure we term iBIC (for ‘integrated BIC’) which captures how well each model explains the data given how complex it is [58]. The smaller this number, the greater the model parsimony. The difference between two such values, *Δ*iBIC, is an approximation of the models’ relative log Bayes factor.

The same principles of model comparison also apply to the categorical question whether two groups differ in terms of their parameters. That is, when asking whether group A and B differ in terms of their reward sensitivity *ρ* or in terms of their learning rate *ϵ*, the correct approach is to compute the posterior likelihood of models *incorporating* these hypotheses about group differences. That is, we computed pairs of models for each dataset: in the first model of each pair ${ℳ}_{\rho }$, which allows group differences in *ρ*, participants share a common prior for all parameters except for *ρ*, for which the two groups have separate priors. In the second model ${ℳ}_{\epsilon }$, participants similarly share a prior for all parameters except for *ϵ*. Computing the Bayes factors (*Δ*iBIC) values for these two models relative to each other indicates whether group differences in one or the other parameter provide a more parsimonious account of the *entire* dataset while taking into account the relative flexibility each parameter accords the model and interactions between parameters. The Bayes factors of these models relative to the original model with no group separation, indicate whether the groups significantly differ in either characteristic.

### Regression analyses

After model validation, we first assessed inter-correlations between specific questionnaire measures (AD, BDA, GDD, BDI\A, AA, GDA) and reward sensitivity or learning rate in the entire sample using one multiple linear regression analysis for *ρ* and one for *ϵ* (regstats.m in Matlab V7.14). However, this analysis neglected two aspects of the data: first that the questionnaire data are likely correlated; and secondly that parameters for different participants are estimated with different degrees of confidence. We therefore ran a weighted hierarchical multivariate regression. This is equivalent to a standard hierarchical multivariate regression, except that parameters were weighted by the precisions with which they were estimated (see [60] for details). Note that parameters are represented in the transformed space throughout to avoid issues with non-Gaussianity.

## Results

### Model validation

We built a set of models that embody key hypotheses about the course of learning in the different groups and fitted them to the data. The models parameterize the *type* of learning performed by participants, allowing us to assess whether they attach rewards to stimulus-action pairs; or just to actions, or to a mixture of the two. We also test the status of ‘no reward’: do participants treat this outcome as a punishment, reducing the probability of the associated action, or do they treat it as a non-informative null outcome, as intended?

Since we are interested in understanding the characteristics of groups of individuals, we need to ascertain at the group, rather than at the individual level, which model does best [43,45,58]. We indeed found that, taking suitable account of complexity, a single model did capture all groups satisfactorily, allowing for a common and interpretable semantics for the parameters. These so-called random effects models capture inter-subject variation in a way that allows for differences in the extent to which individual participants’ data constrain the parameters. When we perform correlation analyses (below), we weigh parameters according to the precision with which they were inferred.

**Figure 2:**
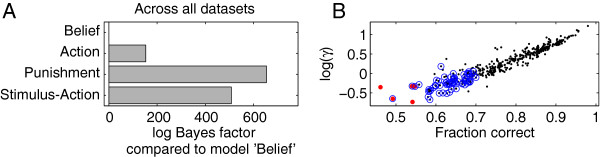
**Model performance.A:** Model comparison. Group-level log Bayes factors *δ*iBIC for each model relative to model ‘Belief’ across all datasets. A difference ≥10 in this measure is strong evidence for the model with the lower score. **B**: The parameter *γ* in the model largely captures the probability with which participants made a correct choice. Note that, by design of the task, this explicitly captures the effect of symmetric instructions and perceptual difficulties, rather than the asymmetric effect of rewards.

The results are shown in Figure 2A. The most parsimonious account of the data was provided by model ‘Belief’. This conclusion rests on the group-level Bayes factors iBIC (see Methods and Additional file 1 for further details). This compares the approximate posterior probability of each model given the data to that of model ‘Belief’. Two key aspects of this quantity are that 1) it punishes overly complex models; 2) it assesses this at the group, rather than the individual level. The higher this number, the poorer the combination of model fit and model simplicity. Differences above 10 are typically considered to be strong evidence for one model over the other [43].

Both the standard Rescorla-Wagner model ‘Stimulus-action’ with separate stimulus-choice values Q(s,a), and model ‘Punishment’, which treats trials on which no reward was given as punishing, performed poorly with much worse iBIC values. This suggests that participants were not able to treat the two stimuli as entirely separate; and that they did not treat the absence of rewards as punishments with aversive properties. Model ‘Action’, which assumes that participants learn only action values, and thus do not separate between stimuli at all in the asymmetric component of choice (*ζ*=0.5) was an improvement over the basic model ‘Stimulus-action’ (*ζ*=1) but came a distant second to the model ‘Belief’ (*ζ* inferred for each session). Indeed, the belief parameter *ζ* was broadly distributed around 0.5 (mean 0.53, standard deviation 0.13). That is, on average, participants behave as model ‘Action’, neglecting the stimuli when learning and forming expectations. Individually, however, there was variability in how well they were able to discriminate the stimuli.

Figure 1F shows the probability of performing a correct choice for all participants in all datasets, with those not predicted better than chance by model ‘Belief’ circled in blue. This model correctly predicted significantly more choices than chance (binomial test, *p*<.05) for most experimental sessions (329 out of 392; black dots). Due to a smaller difference between long and short mouth, and hence a more difficult perceptual discrimination problem (Table 1), participants in the stress dataset– whether in the stress or the no stress condition–showed on average a lower probability of correct choice. They were consequently less well predicted by all models, but model ‘Belief’ still gave the best account of these data too (see Additional file 1: Section S2.1). Figure 2B shows that, as intended, the overall probability correct was captured by the instruction sensitivity parameter *γ* (Pearson correlation 0.93, *p*<10^−20^), which in turn frees the other parameters to capture trial-to-trial variation in the behaviour contingent on the reward outcomes.

Finally, Additional file 1: Section S2.2 also provides the results of a resampling analysis. This shows that if the model is run on the task, it spontaneously generates data that looks similar to the experimental data.

### Regression analyses

Given that model ‘Belief’ captured the data satisfactorily, we proceeded to analyse the relationship between model parameters and self-report questionnaire measures. Our aims were primarily to identify correlations between measures of anhedonia and learning rates or reward sensitivities. A standard, unweighted, multiple linear regression analysis revealed a significant negative correlation between *ρ* and anhedonic depression (AD), the most specific measure of anhedonic symptoms available to us (*p*=0.004; uncorrected for multiple comparisons). There was no correlation between *ρ* and other questionnaire measures, and no correlation between the questionairre measures and the learning rate *ϵ* (all *p*>0.1). Participants in the ‘Stress’ dataset were tested twice. Repeating this analysis using only the session without stress yielded similar results (*p*=0.007 for the correlation *ρ* vs AD, all other *p*>0.09).

**Figure 3:**
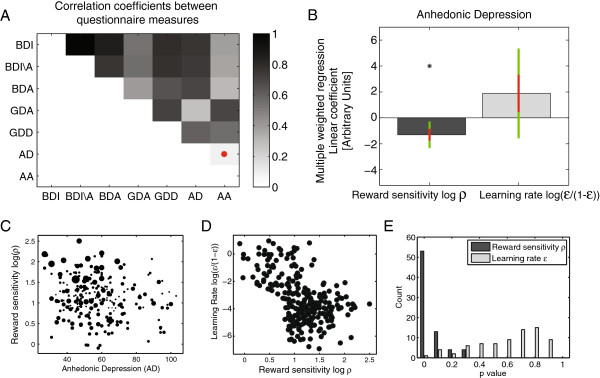
**Correlates of anhedonia.A:** Correlation coefficients for all pairwise correlations between questionnaire measures. All are highly significant (*p*<.01), except for the correlation between anhedonic depression and anxious anxiety, denoted by a red dot. **B**: Hierarchical weighted regression analysis across all datasets, involving all 255 participants with a full set of BDI, BDA and MASQ scores. The plots shows the linear coefficients between anhedonic depression (AD) score and the reward sensitivity and learning rate parameters *ρ* and *ϵ*. Each bars shows one linear coefficient; the red error bars indicate ± 1 standard error; and the green error bars indicate the 99.4% confidence interval (corresponding to a Bonferroni corrected level *p*=.05/8). AD is significantly and negatively correlated with the reward sensitivity *ρ*, but not significantly correlated with the learning rate *ϵ*. **C**: Scatter plot of anhedonic depression against reward sensitivity. Size of dots scale with weight (inference precision). **D**: Scatter plot of reward sensitivity vs. learning rate. **E**: Significance of correlations across parameter estimates from 70 surrogate datasets. There is a consistent and stably significant correlation between AD and reward sensitivity *ρ*, but not between AD and learning rate *ϵ*.

As expected, questionnaire scores were substantially correlated (Figure 3A), and the parameters of different experimental sessions were inferred with varying certainty. We therefore additionally orthogonalized the three anhedonic measures (BDA, GDD, AD) with respect to all the three other measures (BDI\A, AA, GDA) and fit a weighted generalised linear model (GLM). Figure 3B shows that anhedonic depression AD remained significantly and negatively related to the reward sensitivity *ρ* (*p*=0.005, Bonferroni-corrected for 8 comparisons). No other correlation survived correction for multiple comparisons (all *p*>0.1). In particular, no measure of anhedonia was associated with the learning rate *ϵ*. Figure 3C shows a scatter plot of AD scores vs. reward sensitivity with dot size proportional to the weight (inverse variance) in the weighted regression analysis.

Next, there was a negative linear correlation between *ρ* and *ϵ* of -0.41 (*p*<0.0001; Figure 3D). To further question the selectivity of the correlation between AD and *ρ*, we orthogonalized *ρ* with respect to *ϵ* in addition to orthogonalising as above. This again yielded an (unweighted) significant correlation of AD with *ρ* (*p*=0.004, multiple weighted linear regression, uncorrected for multiple comparisons), with no other correlations significant. The reverse orthogonalization did not yield any significant correlations with *ϵ*.

At least part of the correlation between *ρ* and *ϵ* arises because the the two parameters can explain similar features of the data, i.e. alterations in one parameter can be compensated for by alterations in the other parameter (see Figure 1). To establish whether the association between AD and the reward sensitivity parameter was due to real features in the data, rather than due to inference issues, we asked whether the correlations with questionnaire measures remained stable and identifiable in the surrogate data. For each of the 70 surrogate datasets of 392 experimental sessions, we repeated the standard, unweighted multiple linear regression. Figure 3E shows the distribution of *p* values for the correlation of AD with *ρ* and *ϵ*. While the median *p* value for the correlation between *ρ* and AD was 0.04, that for *ϵ* and AD was 0.71.

Finally, all correlation analyses using the reward sensitivity and learning parameters inferred from the second-best model ‘Action’ yielded the same results, showing that the results are not dependent on a particular model formulation.

### Categorical comparisons

We next examined how learning rate and reward sensitivity were affected by the factors explored in each of the individual datasets. For each dataset, we compared two models: one which assumes that the two experimental groups differed in terms of *ρ*, the other in terms of *ϵ*.

**Figure 4:**
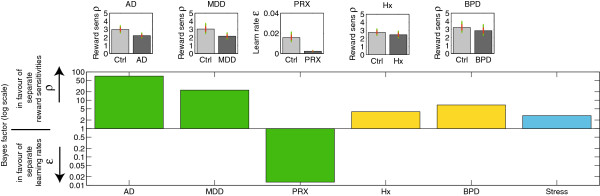
**Comparing models incorporating hypotheses about group differences in terms of reward sensitivity*ρ* or learning rate*ϵ*.** Each bar of the large panel shows the Bayes factor comparing _*ρ*_ to _*ϵ*_. Green bars indicate very strong evidence for model _*ρ*_ (Bayes factor ≥20), yellow bars weak evidence (Bayes factor 3−10) and cyan bars insufficient evidence (Bayes factor <3). MDD and high scores of anhedonic depression (AD) result in a reduction in *ρ*. Pramipexole instead reduces the learning rate. Stress has no differential effect on reward sensitivity compared with learning rate. Participants with a past history of depression, or euthymic individuals with bipolar disorder, showed weak evidence for a reduction in *ρ*. The insets at the top show the inferred priors over the relevant parameters of the winning model for each group.

Figure 4 shows the Bayes factors for models ${ℳ}_{\rho }$ compared with ${ℳ}_{\epsilon }$ for each of the individual datasets. Given the correlation results, we additionally performed a comparison between all participants with the 20% highest and lowest AD scores. Bayes factors above 20 (or below 1/20) are very strong evidence in favour of a hypothesis [43], while likelihood ratios of 3 to 10 (or the inverse) are weak evidence. We found strong evidence that MDD and AD were better accounted for by a change in reward sensitivity *ρ*, rather than learning rate *ϵ*. The opposite was true for pramipexole, which mainly acted by reducing the learning rate *ϵ*.

However, the Bayes factors comparing models ${ℳ}_{\rho }$ and ${ℳ}_{\epsilon }$ to the basic ‘Belief’ model without split parameters were all ≤3. This means that there was no strong evidence that either of these parameters categorically separates any two groups. Stress appeared most likely to have a genuinely shared effect on both parameters. Participants with a history of depression, or currently euthymic bipolar participants showed weak evidence of reductions in reward sensitivity. Note that all these ratios are by necessity numerically more modest than those in Additional file 1: Figure S1 because they are inferred from far less data.

## Discussion

Our results suggest that anhedonia (as measured by AD) and MDD affect appetitive learning more by reducing the primary sensitivity to rewards *ρ* than by affecting the learning rates. Non-significant trends for such an effect were observed for participants with a past history of depression, and amongst euthymic bipolar disorder patients. By contrast, a dopaminergic manipulation was found to preferentially affect the speed of learning, *ϵ*. Acute stress had no preferential effect on reward sensitivity or learning rate. These two parameters appear to be state, rather than trait measures: Neither a past history of depression, nor one of BPD, had a significant effect on *ρ* or *ϵ*.

### Anhedonia

Two self-report measures of anhedonia were used in this paper: the anhedonic depression subscore of the MASQ questionnaire, and the anhedonic subscore of the BDI. The former was clearly related to the reward sensitivity *ρ*. This subscore quantifies participants’ verbally expressed inability to experience pleasure, and so might be expected to capture something akin to consummatory ‘liking’ [40]. Note, however, that various studies (e.g. [18,61,62]) have failed to find a direct correlate between anhedonia measures such as those employed here and participants’ ratings of how pleasant a sucrose solution is—a widely-used animal model of anhedonia [63].

By contrast, in our paradigm, *ρ* falls squarely within the behavioural definition of ‘wanting’ whereby past experiences are collated into values that influence choice. One possibility is thus that our results reflect the way that ‘liking’ is coupled into ‘wanting’, with the reward having less impact when it occurred (*ρ* was lower), rather than the same amount of pleasure not being translated efficiently into anticipation (which would be the consequence of lowered *ϵ*). Such a reduction in reward sensitivity parallels reductions in emotional reactions seen in MDD in response to appetitive, but also aversive, stimuli [64].

At a neurobiological level, ‘liking’, has been linked to *μ*-opioid signalling [40,65-67] in NAcc shell. Recent PET studies have reported prefrontal changes in *μ*-opioid receptors in MDD [68], and *μ*-opioid receptors have been found to affect the response to antidepressant medications [69]. We also note that substance abuse disorders, including of opiates, are prominently co-morbid with depressive disorders [70,71], although it is not clear whether abuse causes depression or *vice versa*. It would be interesting to examine the effect of opiates on tasks like those examined here. If the primary reward sensitivity relates to “liking” and consummatory pleasure, then one would expect a preferential impact on *ρ*, unlike dopamine.

### Dopamine in depression

The temporal difference learning model of phasic dopamine signalling posits that DA reports the prediction error *δ*. As mentioned in the introduction, a multiplicative change in this signal would be equivalent to a change in the learning rate *ϵ*. Although an alteration in the reward sensitivity *ρ* necessarily also leads to an alteration in the prediction error *δ*, the consequences of an abnormality that arises upstream of the prediction error are subtly different from an abnormality affecting the already computed prediction error signal. It is the latter that is most closely aligned with a primary alteration in dopaminergic function.

In the current study, we report tentative findings suggesting that pramipexole reduced the learning rate rather than affecting the reward sensitivity, which might correspond to a direct reduction in the signal reported by phasic DA. Although pramipexole is a non-ergot D _2_/*D*_3_*agonist* (and is clinically used as such in Parkinson’s disease, restless leg syndrome, and occasionally in treatment-resistant MDD), it has previously been found to have behavioural effects of a DA *antagonist* at the low doses (0.5 mg) used in our dataset [72-74]. Similarly, low doses of the D _2_ agonist cabergoline have been found to specifically reduce reward go learning [75]. With both drugs, it has been postulated that this may be due to activation of inhibitory [76] presynaptic D _2_ receptors, which have a higher affinity for DA [77] and *reduce* phasic DA release ([78-82]; see also [56]) and DA cell firing [83]. Thus, our findings echo those of a recent study showing that dopamine agonists can reinstate prediction errors by restoring the component of the prediction error related to expected value, rather than that of reward [84]. However, we emphasize that the conclusions pertaining to dopamine rest on the contribution of only one study and that there are important technical caveats to be borne in mind (see below).

As indicated in the introduction, DA has multiple and profound involvements with depression. These range from the fact that DA manipulations affect mood [85] and that many antidepressants have pro-DA effects (including buproprion, sertraline, nomifensine, tranylcypromine amongst others; [86]), to a role in resilience [87,88], and suicidality in MDD [89,90]. Critically, depression is common in other disorders that also involve DA dysfunction, such as Parkinson’s disease and schizophrenia [91,92]. However, the findings reported here speak to the specific aspects of phasic DA in learning, rather than to all the manifold ways in which DA may be involved in anhedonia and depression. Tonic DA levels, which are partially independent of phasic bursts of activity [93,94] have a profound impact on energy levels and vigour [95,96] making this feature of DA a likely candidate for the severe psychomotor retardation seen in melancholic depression [32], and to the distinction between pleasure and motivational aspects of anhedonia [18,97]. In the context of our task, this may relate to reaction times (albeit noting that these can also be affected by phasic DA signals; [98]), which were not analysed. Rather, here, we focused on the role of DA in instrumental learning of action values. Note, though, that the current design cannot completely disentangle instrumental effects from those due to Pavlovian approach [58,99]. Such Pavlovian influences involve dopamine and the nucleus accumbens (NAcc; [100-105]), and may indeed constitute one of the ways in which DA supports antidepressant function [106,107].

Overall, our findings are consistent with the fact that DA by itself is not a major target for psychopharmacological treatment of anhedonia [92], and, modulo the issues mentioned above, that DA appears to mediate ‘wanting’, more than ‘liking’ [40,108]. There has been a number of recent functional imaging investigations into reward-related decision making in MDD (e.g. [11,109-114]). Our findings have key implications for the interpretations of these studies, as it is important to separate contributions to the correlates of prediction errors that arise from changes in reward sensitivity from changes in learning rate. Because a) ‘wanting’ (reinforcement) and ‘liking’ (hedonic) aspects of tasks will often overlap; and b) both changes in *ρ* and *ϵ* would affect correlations with a regressor based on prediction errors *δ*, our findings do not invalidate these imaging results, but call for studies and analyses that further separate them.

### Analysis methods

Our conclusions were derived from a detailed, model-based meta-analysis combining behavioural data from six datasets in 392 sessions. Several features and limitations of this analysis deserve comment.

First, the interpretability of the parameters is maximised by using rigorous model comparisons. The Bayesian approach we used prevents overfitting by *integrating out* individual subject parameters [43,45,46,58]. Rather than just comparing how careful parameter tuning can allow a model to fit the data extremely well, we extensively sampled the model’s entire parameter space, and ask how well, on average, the *class* of model, independent of its particular parameters, can fit the given data (for an insightful explanation, see chapter 28 of [59]). Next, our model comparison is done at the group level, rather than at the individual level, again through sampling and Bayesian model comparison. This ensures that conclusions about the parameters do apply to the group.

Second, our approach takes individual model fits into account. The regression analyses are true random effects analyses, weighting parameters by how strongly they are constrained by each participant’s own choice data, and by how well the model fits that particular participant. This, in combination with the explicit modelling of stimulus uncertainty (beliefs) and instruction weights, ensures that any non-specific performance variability does not unduly affect our parameters of interest. Furthermore, the weighted regression ensures that each participant influences the conclusions proportionally to how well they are fit by the model.

Third, it is standard practice to constrain the parameters when fitting models, for instance to avoid extreme outlier inference. We use two types of constraints. The parameter transformations generate hard constraints that force parameters to remain inside feasible regions. The empirical Bayesian inference of the group priors additionally yields the most appropriate soft constraints [59,115].

Fourth, learning rate and reward sensitivity were correlated in all models tested. To alleviate this, we enforced independence at the group level. One standard approach would have been to compare models in which one dimension is constrained by forcing the parameters for all participants along that dimension to be equal. However, this a) is an unrealistic constraint; b) fails to address the fact that parameters may not give the model equal flexibility; and c) renders parameters hard to interpret as variability from one is squeezed into all the other parameters (which further aggravates point b). To circumvent these issues, we contrasted the parsimony of models that explicitly allowed participants to fall into distinct groups. Doing this at the group level addressed the questions at the group level, which is where we sought to draw conclusions. We also performed the regression analysis in a number of ways to assess the selectivity of the relationship with *ρ* and its stability in the inference procedure.

Fifth, it is important to note that our failure to discover correlations between AD and learning rate, or between the effects of pramipexol and reward sensitivity might be due to limits of power. This is particularly true for the categorical comparisons on which arguments about the differential effect of dopamine and anhedonia or depression rest mainly. It will be critical to replicate these findings in larger samples, potentially requiring paradigms explicitly adapted to separating the two factors.

Sixth, we find no evidence that either learning rate of reward sensitivity clearly separates any of the groups when comparing the basic model to models ${ℳ}_{\rho }$ or ${ℳ}_{\epsilon }$ that allow for the two groups to have different means in one of the other parameter. However, our central motivation was whether anhedonia associated more with unusual *ρ* or unusual *ϵ*, because of the different psychiatric and psychological interpretations of these possibilities. The most direct test of this is to compare ${ℳ}_{\rho }$ to ${ℳ}_{\epsilon }$ rather than comparing each to a mid-point in the shape of the basic model. Furthermore, there are a number of caveats to this finding, which form the basis for reporting the more lenient comparison between models ${ℳ}_{\rho }$ and ${ℳ}_{\epsilon }$. First, the model comparison technique may be too conservative for this particular analysis. For instance, at the top level we very stringently punish according to the total number of observations. Although we have found this to overestimate the rate at which the group-level variance should drop with observations, in our hands this penalty has proven to yield the correct responses most reliably when tested on a variety of surrogate datasets (unpublished data in preparation). This is likely to be particularly important given that the power in the analyses in Figure 4 is much lower than that in the preceding analyses using the entire dataset. These considerations do not bear on comparisons between models ${ℳ}_{\rho }$ and ${ℳ}_{\epsilon }$. Furthermore, the panels showing the parameters in Figure 4 appear to show robust group differences. However, a separation in parameter space does not guarantee that the increase in fit outweighs the increase in model complexity.

Of course, even though our best fitting model did an excellent job predicting the data of a plurality of participants, there could be a model that we did not try that would do even better. This is particularly true of the participants in the Stress dataset, who were fit the worst. There is a conventional ANOVA-like procedure in these circumstances that involves assessing the extent to which responses are potentially predictable [44,116] from running the same task multiple times. Unfortunately, it is not clear how to execute this in cases involving learning which is contingent on the participants’ behaviour.

### Alternative models

One advantage of the simplicity of the task is that it is likely insensitive to several aspects of reinforcement learning that are under current investigation, such as goal-directed *versus* habitual decision-making [117,118]. However, one interesting direction would be to consider a more Bayesian treatment of the learning process, according to which the effective learning rate *ϵ* should be a function of the amount of experience that the participant has had, modulated by the participant’s belief that the contingencies are constant [119-121].

This elaborate account raises an important question about the factor *ϵ* in the models that we did build. Here, we considered the effect on learning of manipulating the magnitude of *δ*_*t*_; but noted that this is exactly confounded in the magnitude of *ϵ*. Neurobiologically, though, there could be an alternative realization of *ϵ*, notably via cholinergic influences [122-124]. It would be interesting to examine the effect of cholinergic manipulations on the basic task.

Equally, in conventional reinforcement learning models, it is common to employ a variant of Equation 2 in which the terms W are multiplied by another arbitrary constant which is usually written *β* and called an inverse temperature. The larger *β*, the more deterministic the choice between *a*_*t*_ and ${ā}_{t}$, all else being equal. Thus *β* is often used as a surrogate for controlling exploration, as more stochastic choices (lower *β*) are more exploratory. However, in our model, *β* can substitute *exactly* for *ρ* (in a very similar way that *ϵ* can substitute for the magnitude of *δ*). Thus reward insensitivity could masquerade as over-exploration or (particularly in depression) as under-exploitation. These are not differentiable in this task. Nevertheless, the neurobiological realization of the exploration constant *β* has been suggested as being rather different – notably involving noradrenergic neuromodulation [125] – opening up another line of experimental investigation.

Finally, two relevant findings may be important for future model development. First, positive affective responses to positive events in daily life have recently been found to be *stronger* in depressed than in non-depressed individuals [126]. Second, the impact of negative events has been found to be determined not by the strength of the immediate emotional reaction, but by the attributions made about it [127]. Thus, it may be that the effects seen here can be modified by higher-order processes in ways that are critical for the development of psychopathology, and it may be useful to extend the present methods to such interactions [60].

## Conclusions

This paper presented a model-based meta-analysis of behavioural data spanning several related manipulations and adds to a growing literature of behavioural correlates of depression [64,128]. We concluded that anhedonia in depressive states was mediated by a change in reward sensitivity, which has different behavioural consequences from either stress or DA manipulations. Our analysis allowed us to draw these conclusions while taking into account as much as possible the variability between the datasets and participants; and the variability in other, unrelated aspects of task performance. We believe that similar analyses could be readily applied to other datasets. We hope that our findings will encourage the re-analysis of the dissociation between reward sensitivity and dopaminergic processes in depressive states.

## Competing interests

The authors declare that they have no competing interests.

## Authors’ contributions

QJMH and PD performed the computational modelling analysis. DAP and RB devised the experiment and collected the data. All authors wrote the manuscript. All authors read and approved the final manuscript.

## Supplementary Material

Additional file 1 **Supplementary methods and results [**[43]**,**[63]**-**[65]**].** Click here for file
