# Supplementary material for: Mapping anhedonia onto reinforcement learning: a behavioural meta-analysis
Source: Biol Mood Anxiety Disord. 2013 Jun 19;3:12. doi: 10.1186/2045-5380-3-12 (PMC3701611; doi:10.1186/2045-5380-3-12)
Supplement: Additional file 1 — Supplementary methods and results [[43],[63]-[65]]. [file 2045-5380-3-12-S1.pdf]

1 Supplementary Methods

1.1 Fitting procedures

Our Bayesian model fitting and comparison approach has been described in detail previously [58,60], but we repeat it here very briefly for completeness.

Each model contains, for each participant, a vector of parameters $\mathbf{h}$. The maximum a posteriori estimate of each parameter $\mathbf{h}_i = \text{argmax}_{\mathbf{h}}\, p(\mathbf{A}_i|, \mathbf{h})p(\mathbf{h}|\boldsymbol{\theta})$ is inferred for each participant i based on that participants' actions $\mathbf{A}_i$. The parameters of the prior distribution $\boldsymbol{\theta}$ are inferred via approximate Expectation-Maximisation [59] and set to the maximum likelihood given all the data by *all* the N experimental sessions:

$$\hat{\boldsymbol{\theta}}^{ML} = \underset{\boldsymbol{\theta}}{\text{argmax}}\, p(\mathcal{A}|\boldsymbol{\theta})$$

Here we let $\mathcal{A} = \{\mathbf{A}_i\}_{i=1}^N$ be all the actions in all the experimental session. The approximation concerns the use of a Laplacian approximation for the E-step, letting:

$$\begin{aligned} p(\mathbf{h}|\mathbf{A}_i) &\approx \mathcal{N}(\mathbf{h}_i^{(k)}, \Sigma_i^{(k)}) \\ \mathbf{h}_i^{(k)} &= \underset{\mathbf{h}}{\text{argmax}}\, p(\mathbf{A}_i|\,\mathbf{h})p(\mathbf{h}|\boldsymbol{\theta}^{(k-1)}) \end{aligned}$$

$\mathcal{N}(\cdot)$ denotes a Gaussian distribution with variance $\Sigma_i^{(k)}$ and mean $\mathbf{h}_i^{(k)}$. In the M-step, the group level mean parameters $\mathbf{m}$ and (factorized) variance $\mathbf{v}^2$ are inferred simply as:

$$\begin{aligned} \mathbf{m}^{(k)} &= \frac{1}{N}\sum_i \mathbf{h}_i^{(k)} \\ (\mathbf{v}^{(k)})^2 &= \frac{1}{N}\sum_i \left[(\mathbf{h}_i^{(k)})^2 + \Sigma_i^{(k)}\right] - (\mathbf{m}^{(k)})^2 \end{aligned}$$

1.2 Model comparison

Models are compared based on the likelihood of the data given the model $\log p(\mathcal{A}|\mathcal{M})$ because we have no *a priori* preference for any particular model. As previuosly, we approximate this quantity in two steps. At the group level [43], we utilize the standard Bayesian Information Criterion (BIC) approach:

$$\begin{aligned} \log p(\mathcal{A}|\mathcal{M}) &= \int d\boldsymbol{\theta}\, p(\mathcal{A}|\boldsymbol{\theta})\, p(\boldsymbol{\theta}|\mathcal{M}) \\ &\approx -\frac{1}{2}\text{iBIC} = \log p(\mathcal{A}|\hat{\boldsymbol{\theta}}^{ML}) - \frac{1}{2}|\mathcal{M}|\log(|\mathcal{A}|) \end{aligned}$$

Here, $|\mathcal{A}|$ is the total number of choices summed over all experimental sessions while $|\mathcal{M}|$ is the number of prior parameters fitted (note that there are one group-level mean and one group-level variance for each parameter).

Notably, though, $\log p(\mathcal{A}|\hat{\boldsymbol{\theta}}^{ML})$ is the sum of *integrals* over the individual parameters (hence the prescript "i" to BIC). We evaluate this by sampling from the empirical prior and averaging over the samples:

$$\begin{aligned}\log p(\mathcal{A}|\hat{\boldsymbol{\theta}}^{ML}) &\approx \sum_i \log \frac{1}{K}\sum_{k=1}^{K} p(\mathbf{A}_i|\mathbf{h}^k) \\ \mathbf{h}^k &\sim p(\mathbf{h}|\boldsymbol{\theta}^{ML})\end{aligned}$$

1.3 Belief model specification

The model 'Belief' applies the belief variable ζ only to the learned $\mathcal{Q}$ values, and not to the instructions $\mathcal{I}$. Prima facie this is surprising, as if a subject is able to correctly identify the stimulus to follow instructions, then they should be able to do so for the learned values, too. Here we briefly show that the models are, in fact, equivalent.

In model 'Belief', choices are determined by the term in the softmax:

$$\mathcal{W}_t(a_t, s_t) - \mathcal{W}_t(\bar{a}_t, s_t) = \gamma(\mathcal{I}(a_t, s_t) - \mathcal{I}(\bar{a}_t, s_t)) + f(\mathcal{Q})$$

where $f(\mathcal{Q})$ is a term that only involves $\mathcal{Q}$ and so is not important for the effect of ζ on $\mathcal{I}$.

An alternative model, where ζ applies to both the learned component $\mathcal{Q}$ and the instructions $\mathcal{I}$, would read:

$$\begin{aligned}\mathcal{W}_t(a_t, s_t) =&\gamma(\zeta\mathcal{I}(a_t, s_t) + (1-\zeta)\mathcal{I}(a_t, \bar{s}_t)) + \\ &\zeta\, \mathcal{Q}_t(a_t, s_t) + (1-\zeta)\, \mathcal{Q}_t(a_t, \bar{s}_t)\end{aligned}$$

and so the term in the choice-determining softmax would be

$$\begin{aligned}\mathcal{W}_t(a_t, s_t) - \mathcal{W}_t(\bar{a}_t, s_t) =&\gamma(\zeta\mathcal{I}(a_t, s_t) + (1-\zeta)\mathcal{I}(a_t, \bar{s}_t)) - \\ &\gamma(\zeta\mathcal{I}(\bar{a}_t, s_t) + (1-\zeta)\mathcal{I}(\bar{a}_t, \bar{s}_t)) + f(\mathcal{Q})\end{aligned}$$

however, $\mathcal{I}(a_t, s_t) = \mathcal{I}(\bar{a}_t, \bar{s}_t)$, and similarly for the off-diagonal terms of $\mathcal{I}$, so we have

$$\mathcal{W}_t(a_t, s_t) - \mathcal{W}_t(\bar{a}_t, s_t) = \gamma((2\zeta - 1))\mathcal{I}(a_t, s_t) - (2\zeta - 1)\mathcal{I}(\bar{a}_t, s_t) f(\mathcal{Q})$$

which is this equivalent to redefining γ as $\gamma(2\zeta - 1)$.

Another alternative, would be to argue that uncertainty affects not only the selection of the appropriate $\mathcal{Q}$ value, but also the learning. To test this legitimate idea, we fitted an additional model in which, on each

iteration, the $\mathcal{Q}$ value of the chosen action a_t for both the presented stimulus s_t and the other stimulus $\bar{s}_t$ is updated to an extent proportional to the belief:

$$\begin{aligned}\mathcal{Q}(a_t, s_t) &= \mathcal{Q}(a_t, s_t) + \zeta\epsilon(\rho r_t - \mathcal{Q}(a_t, s_t)) \\ \mathcal{Q}(a_t, \bar{s}_t) &= \mathcal{Q}(a_t, \bar{s}_t) + (1-\zeta)\epsilon(\rho r_t - \mathcal{Q}(a_t, \bar{s}_t))\end{aligned}$$

This model performed very similarly to the model 'Action' and performed worse than the model 'Belief' (log Bayes factor compared to model 'Belief' 138.04). We therefore did not analyse it further.

2 Supplementary Results

2.1 Model comparison for Stress dataset

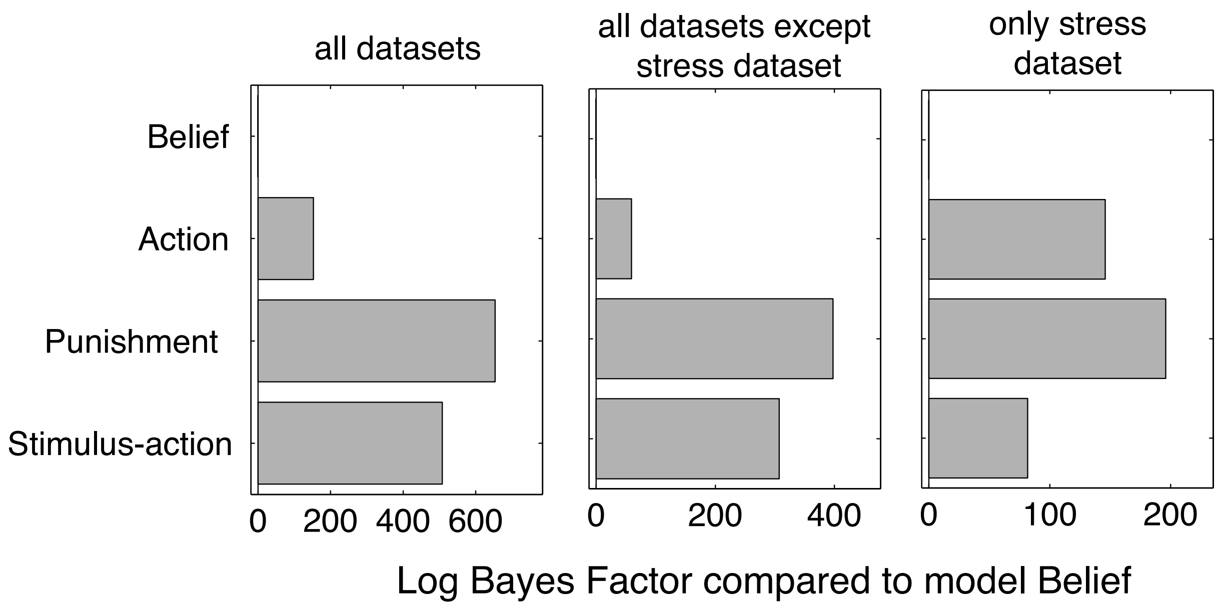

Figure S1: Model comparison log Bayes factors. Left panel is the same as in Figure 2A for comparison; the middle panel for all but the Stress dataset; and the right panel for the Stress dataset alone. Throughout, there is clear evidence for model 'Belief'.

Because they faced a more difficult perceptual task (a smaller difference between the 'long' and the 'short' mouth), participants in the stress dataset, showed on average a lower probability of correct choice, and were consequently less well predicted by all models (c.f. Figure 2). We therefore performed additional, separate model fits and comparisons for the Stress dataset, and the remaining five datasets; and for all datasets separately. Figure S1 shows the results for the Stress dataset and all other datasets combined. The same 'Belief' model provided the (jointly) best fit in all datasets. In addition, the belief values did not differ between the Stress dataset and the other datasets (data not shown). Combined with the finding that participants who responded correctly less often had lower γ values (Figure 2C), this shows that these participants specifically assigned less weight to the instructions, but not that they differed in their ability to identify, or learn about, the stimuli. Thus, the accuracy difference between the Stress dataset and the other datasets was well captured by the parameter γ of the model and does not require separate treatment of the Stress dataset.

2.2 Surrogate data comparison

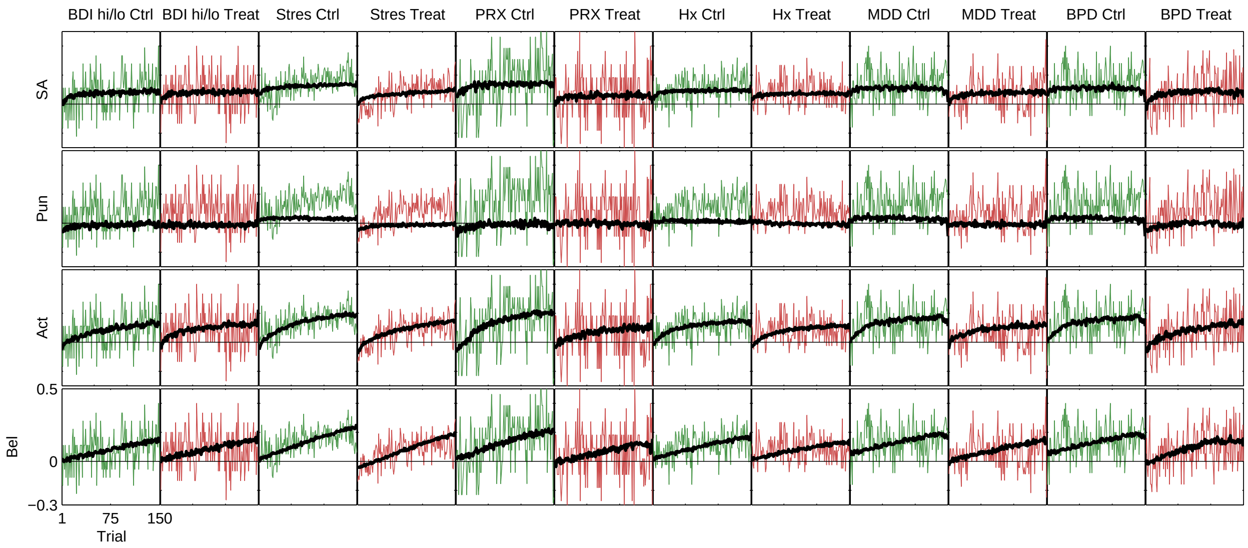

Figure S2: Evolution of choice bias for all datasets (left to right) and all models (top to bottom). Each dataset is split into two panels, showing the 'control' and 'treatment' subgroups in green and red, respectively. Each curve shows the difference in the log probability of a correct response to the rich minus the lean stimulus, averaged across participants. The black curves show the same measure averaged across the 70 surrogate datasets. Note that these models were fit to all six datasets jointly, yet all datasets are fitted reasonably well.

As additional test of model fit, we generated data from the model. Sampling is a good guide as to whether the model captures the data; but does not measure parsimony. We generated 70 surrogate datasets from each models for direct comparison with the experimental results on the evolution of the choice bias log p(correct response—rich stimulus) - log p(correct — lean stimulus). We obtained the *maximum a posteriori (MAP)* parameters $\mathbf{h}^k$ for each participant, and then used this together with the original sequence of stimuli for that participant to generate a novel sequence of 300 choices, although we used a truly random reward sequence rather than the pseudorandom sequence used in the experiment to generate this data. Because datasets were pooled for parameter inference, we expect the resampled data to regress towards the mean of all datasets (this effect should be stronger in smaller datasets). However, we should expect roughly to replicate the general pattern observed in the raw data.

Figure S2 shows the evolving bias towards the rich stimulus for all models and all datasets. The evolution of the bias was reasonably well captured across all datasets. It is notable that the model 'Action' seems to perform similarly to the model 'Belief' despite being simpler. We therefore repeated the regression analyses with the parameters derived from this model.
